# Angiography‐Derived Fractional Flow Reserve: More or Less Physiology?

**DOI:** 10.1161/JAHA.119.015586

**Published:** 2020-03-11

**Authors:** Paul D. Morris, Nick Curzen, Julian P. Gunn

**Affiliations:** ^1^ Department of Infection, Immunity and Cardiovascular Disease University of Sheffield United Kingdom; ^2^ Department of Cardiology Sheffield Teaching Hospitals NHS Foundation Trust Sheffield United Kingdom; ^3^ Insigneo Institute for In Silico Medicine University of Sheffield United Kingdom; ^4^ Coronary Research Group University Hospital Southampton NHS Foundation Trust Southampton United Kingdom; ^5^ Faculty of Medicine University of Southampton United Kingdom

**Keywords:** computational flow dynamics, computer‐based model, coronary microvascular resistance, fractional flow reserve, imaging, Coronary Circulation, Translational Studies, Angiography, Revascularization

Evidence robustly demonstrates that ischemia, rather than anatomy, is the optimal target for coronary revascularization. In the cardiac catheter laboratory, fractional flow reserve (FFR) and corresponding diastolic indices are regarded as the gold standard for physiological lesion assessment and ischemia detection (Table 1). Yet, despite a wealth of supporting data and indications in international guidelines, the use of FFR remains surprisingly low in the diagnostic assessment of coronary artery disease across the world.^1^, ^2^ To address this, multiple groups have developed methods for computing FFR from invasive angiography, without the need for passing a pressure wire or inducing hyperemia, thus removing the main barriers to uptake. Angiography‐derived FFR therefore has the potential to extend the benefits of physiological coronary lesion assessment to considerably more patients. Given the size of the interventional cardiology market, clinical and commercial motivation to deliver these tools as quickly as possible could hardly be greater. Several models are now approved as medical devices. Imminently, physicians and healthcare providers will have to decide whether to use these tools. But do they truly deliver physiology, and are they accurate enough? There are 3 particular areas of that deserve close scrutiny.

**Table 1:** Angiography‐Based Coronary Physiological Assessment Techniques

Index	Abbreviation	Calculated	Equipment	Potential Benefits	Pitfalls/Limitations
Fractional flow reserve	FFR	Whole cardiac cycle Pd/Pa at hyperemia	Pressure wire	Predicts *percentage* improvement in flow with PCI. Good clinical outcomes data	Does not measure absolute flow and microvascular resistance
Instantaneous wave‐free ratio/resting full‐cycle ratio	iFR/RFR	Pd/Pa during diastolic phase	Pressure wire	Good clinical outcome data, relative to FFR	Does not measure absolute flow and microvascular resistance
Index of myocardial resistance	IMR	(Pd) · (thermodilution derived mean transit time)	Thermo‐ and pressure‐sensitive wire	Microvascular resistance becoming of increasing interest (eg, PCI nonresponders, ANOCA, AMI, HFpEF)	Thermodilution not widely used
Hyperemic microvascular resistance	HMR	Pd/Doppler flow velocity	Doppler and pressure wire	Microvascular resistance becoming of increasing interest (eg, PCI nonresponders, ANOCA, AMI, HFpEF)	Doppler flow velocity challenging to measure. Doppler wires not widely used
Hyperemic stenosis resistance	HSR	(Pa‐Pd)/Doppler flow velocity	Doppler and pressure wire	Objective, direct measure of the resistance of proximal disease	Doppler flow velocity challenging to measure. Doppler wires not widely used. Surrogate index
Angiography‐derived FFR	vFFR/FFR_angio_/QFR	Fluid dynamics equations informed by anatomy	Computational fluid dynamics software	Delivering clinical benefits of FFR without factors that limit the invasive technique	Relatively wide Bland–Altman limits of agreement compared with FFR. Requires excellent angiography. Less accurate in those with nonaverage microvascular resistance
CT‐derived FFR	_CT_FFR	Fluid dynamics equations informed by anatomy	Computational fluid dynamics software (offline)	Reduce the number of unnecessary invasive catheterizations	Relatively wide Bland–Altman limits of agreement compared with FFR
Coronary flow reserve	CFR	(Hyperemic flow surrogate)/(baseline flow surrogate) Flow derived from Doppler velocity or thermodilution mean transit time	Doppler or thermosensitive wire	A surrogate for flow and vasodilatory reserve. Flow more important than pressure, but hard to measure	Prone to same limitations as those for Doppler wire or thermodilution. Variability in baseline measurement can impair interpretation
Absolute coronary flow	Q_b_	Infusion flow · (infusion temp/sensor temp) · 1.08 During continuous saline infusion	Thermosensitive wire, pressure wire, monorail infusion catheter	Predicts absolute (not percentage) coronary flow changes and microvascular resistance	Additional time, expertise, and hardware

All physiological indices are surrogate markers of physiology derived from other measures. AMI indicates acute myocardial infarction; ANOCA, angina and no obstructive coronary artery disease; FFR, fractional flow reserve; HFpEF, heart failure with preserved ejection fraction; MVR, microvascular resistance; Pa, proximal pressure; PCI, percutaneous coronary intervention; Pd, distal coronary pressure; and QFR, quantitative flow ratio.

## Simplification

Methods for computing angiography‐derived FFR are software based. Three‐dimensional arterial anatomy is reconstructed from paired 2‐dimensional angiogram images. Mathematical equations that define hemodynamic laws are then applied to the reconstructed artery to predict the pressure dynamics along the artery, which are displayed as a color‐mapped 3‐dimensional artery. In an effort to rationalize these models to make them practical and expedient for clinical use, many groups have abandoned complex, numerical, computational fluid dynamics simulation in favor of analytical solutions based broadly upon the laws of Bernoulli and/or Poiseuille. These simpler physical laws characterize pressure losses attributable to convective acceleration and viscous friction, respectively. They are quick and simple to execute and perform well under steady (nonpulsatile), laminar flow conditions, in straight conduits. Coronary arteries, however, are not straight, and flow is pulsatile. Furthermore, these laws are unable to accurately characterize complex translesional pressure dynamics, particularly poststenosis pressure recovery, which is the basis of FFR. Some stenosis models make empiric assumptions or corrections for pressure loss and recovery. On average, these may perform adequately, but cannot represent the potentially complex flow patterns in a specific case. Moreover, they may be particularly vulnerable to inaccuracy in the context of serial lesions and diffuse disease in which 3‐dimensional computational fluid dynamics computations more reliably characterize interstenosis hemodynamic interaction. The impact this has on accuracy, in all disease patterns, is yet to be fully determined.

## Assumptions

The discordance between angiographic severity and physiological (FFR) significance is well described and affects ≥30% of lesions. Discrepancies occur because, unlike angiography, FFR elegantly and automatically incorporates the combined and inter‐related effects of coronary flow and microvascular resistance. It is therefore imperative that computational models of angiography‐derived FFR include adequate physiological inputs or “tuning” to represent the maximum blood flow or minimum microvascular resistance; the latter dictates the former, which, in turn, dictates the pressure gradient and FFR. Hemodynamic equations are capable of accurately deriving a variety of physiological parameters, but *only* if other appropriate physiological inputs, such as flow or microvascular resistance, are included. A sensitivity analysis demonstrated that microvascular resistance was *the* dominant influence on angiography‐derived FFR, above and beyond the severity or anatomy of epicardial disease.^3^ Hyperemic flow and minimal microvascular resistance are variable in health and disease and are hard to measure, even with invasive instrumentation. Noninvasive models of angiography‐derived FFR therefore rely upon assumptions about these parameters, or predict them from surrogate markers such as arterial diameter. Again, empiric assumptions may be sufficient overall, for many cases, but will be inaccurate in nonaverage cases with discordant anatomy and physiology, that is, the very cases where FFR is superior to angiography. Therefore, unless models have an accurate method for achieving this, on a patient‐specific basis, the “physiological” prediction becomes simply a function of stenosis geometry and they cannot be a genuine model of FFR at all (Figure). As an example, 1 study of angiographically derived FFR observed a significant reduction in diagnostic accuracy in patients with elevated microvascular resistance.^4^ Paradoxically, physiologically weak models will appear more feasible relative to angiographic appearance, and a potential danger is that user confidence may therefore be increased with poorer methods. FFR has enabled a great stride forward in terms of physiologically guided revascularization. It would be unfortunate if, in an attempt to increase physiological assessment, we were to take half a step back toward assessment based on epicardial arterial anatomy. Table 2 summarizes major trials of angiography‐derived FFR.^4^, ^5^, ^6^, ^7^, ^8^, ^9^, ^10^, ^11^, ^12^, ^13^, ^14^, ^15^, ^16^, ^17^, ^18^

**Figure 1:**
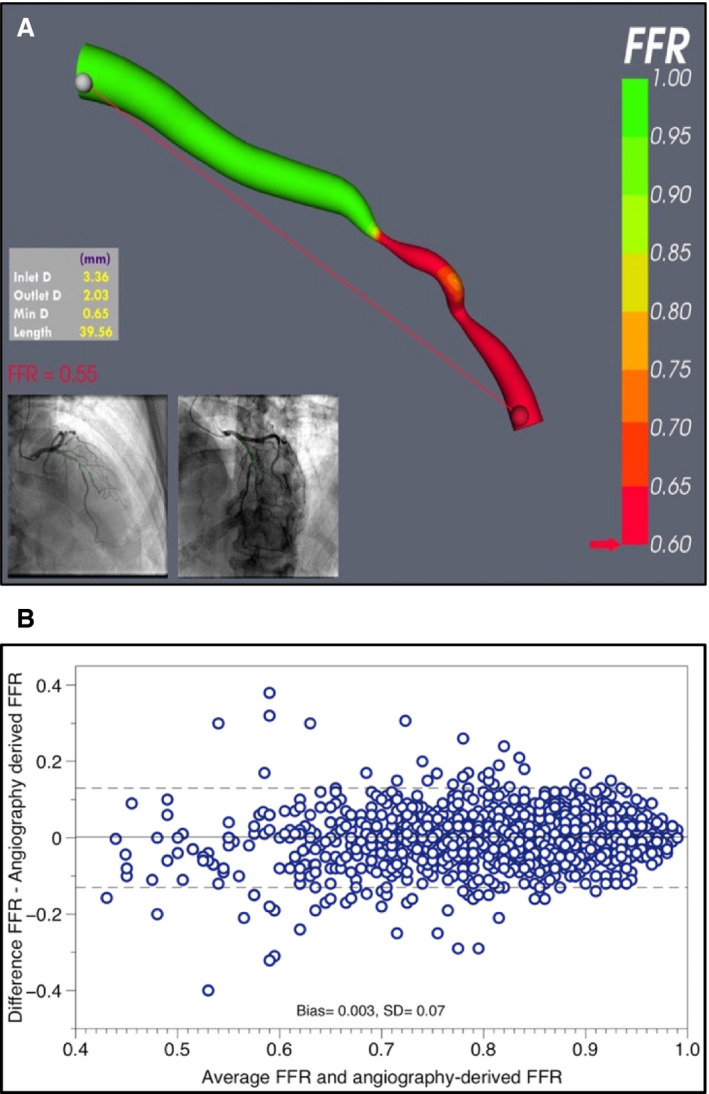
**Error in angiography‐derived FFR.** **(A)** An anatomically severe circumflex case. In this case, the method applied an assumed value for microvascular resistance based on a population average, which resulted in considerable disagreement between angiography‐derived and invasive FFR (0.55 vs 0.82). **(B)** Bland–Altman plot from a meta‐analysis of 13 studies (1842 vessels). There is minimal bias (gray line), but the ±95% limits of agreement were FFR ±0.14. FFR indicates fractional flow reserve. Reprinted from Collet et al^20^ with permission. Copyright ©2018, Oxford University Press.

**Table 2:** Major Trials/Studies of Angiographically Derived FFR

Author	Study	Year	N=Arteries	Surrogate/Software/Company	Mathematical Solution	Diagnostic Accuracy	95% Limits of Agreement
Morris et al^5^	VIRTU‐1	2013	35	vFFR from VIRTUheart (University of Sheffield)	Transient 3D CFD	97%	FFR ±0.16
Tu et al^6^	FAVOUR Pilot	2016	84	QFR from QAngio XA (Medis Medical Imaging Systems, NL)	Empiric flow velocity (fQFR), TIMI frame counting‐derived contrast velocity at baseline (cQFR) and under hyperemia (aQFR). Analytical equations based on laws of Bernoulli and Poiseuille	fQFR 80% cQFR 86% aQFR 87%	FFR ±0.14 FFR ±0.12 FFR ±0.13
Kornowski et al^7^	FFR_angio_ FIM	2016	101	FFR_angio_ (CathWorks, Israel)	Simple analytical equation, based on law of Poiseuille	94%	FFR ±0.10
Trobs et al^8^	FFR_angio_	2016	100	FFR_angio_ from Syngo IZ3D and prototype software (Siemens Healthcare GmbH, Germany)	CFD based on BP, anatomy, and literature estimates of microvascular resistance	90%	FFR ±0.13
Pellicano et al^9^	FFR_angio_ validation	2017	203	FFR_angio_ (CathWorks, Israel)	Simple analytical equation, based on law of Poiseuille	93%	FFR ±0.10
Xu et al^10^	FAVOUR II China	2017	328	QFR from QAngio XA (Medis Medical Imaging Systems, NL)	TIMI frame counting‐derived contrast velocity at baseline (cQFR). Analytical equations based on laws of Bernoulli and Poiseuille	93%	FFR ±0.13
Yazaki et al^11^	QFR in intermediate lesions	2017	151	QFR from QAngio XA (Medis Medical Imaging Systems, NL)	TIMI frame counting‐derived contrast velocity at baseline (cQFR). Analytical equations based on laws of Bernoulli and Poiseuille	88%	FFR ±0.10
Westra et al^12^	WIFI II	2018	240	QFR from QAngio XA (Medis Medical Imaging Systems, NL)	TIMI frame counting‐derived contrast velocity at baseline (cQFR). Analytical equations based on laws of Bernoulli and Poiseuille	83%	FFR ±0.16
Mejía‐Rentería et al^4^	QFR IMR study	2018	300	QFR from QAngio XA (Medis Medical Imaging Systems, NL)	TIMI frame counting‐derived contrast velocity at baseline (cQFR). Analytical equations based on laws of Bernoulli and Poiseuille	IMR <23 =88% IMR ≥23 =76%	FFR ±0.12 FFR ±0.15
Westra et al^13^	FAVOUR II EJ	2018	317	QFR from QAngio XA (Medis Medical Imaging Systems, NL)	TIMI frame counting‐derived contrast velocity at baseline (cQFR). Analytical equations based on laws of Bernoulli and Poiseuille	87%	FFR ±0.12
Fearon et al^14^	FAST‐FFR	2019	319	FFR_angio_ (CathWorks, Israel)	Simple analytical equation, based on law of Poiseuille	92%	FFR ±0.13
Omori et al^15^	FFR_angio_ in multivessel disease	2019	118	FFR_angio_ (CathWorks, Israel)	Simple analytical equation, based on law of Poiseuille	92%	FFR ±0.14
Stahli et al^16^	All comer QFR	2019	516	QFR from QAngio XA (Medis Medical Imaging Systems, NL)	TIMI frame counting‐derived contrast velocity at baseline (cQFR). Analytical equations based on laws of Bernoulli and Poiseuille	93%	FFR ±0.07
Masdjedi et al^17^	FAST‐study	2019	100	vFFR from 3D QCA software, CAAS workstation (PIE Medical Imaging, NL)	Simple analytical equation, based on laws of Bernoulli and Poiseuille	AUC=0.93	FFR ±0.07
Li et al^18^	FLASH‐FFR	2019	328	caFFR from FlashAngio (Rainmed Ltd, China)	CFD based on postangiography TIMI frame counting of flow velocity	96%	FFR ±0.10

Listed in chronological order. Invasive FFR (threshold ≤0.80) was comparator in each study. 3D indicates 3‐dimensional; aQFR, adenosine QFR; AUC, area under the curve; BP, blood pressure; caFFR, coronary angiography–derived fractional flow reserve; CFD, computational fluid dynamics; cQFR, contrast QFR; EJ, Europe and Japan; FFR, fractional flow reserve; FFR_angio_, FFR derived from coronary angiography; FIM, first in man; fQFR, fixed QFR; IMR, index of microcirculatory resistance; QFR, quantitative flow ratio; TIMI, thrombolysis in myocardial infarction; and vFFR, virtual fractional flow reserve.

## Accuracy and Error Range

Headline validation results report “diagnostic” accuracy. This quantifies how well a method predicts physiological significance or nonsignificance (FFR ≤0.80), relative to invasive FFR, expressed as sensitivity, specificity, positive, and negative predictive values, area under a receiver operating curve, and overall diagnostic accuracy. Diagnostic accuracy is a function of (1) the method's accuracy and (2) the cases included in a particular study. The fewer cases close to the 0.80 threshold, the better the diagnostic accuracy will appear and vice versa. This is nicely illustrated in a study of FFR computed from computed tomography coronary angiography in which the diagnostic accuracy was 82% overall, but only 46% in cases in FFR were 0.70 to 0.80, which is precisely the range where most accuracy is required.^19^

The best test of how accurately angiography‐derived FFR agrees with invasive FFR is to plot the differences between predicted and observed FFR values against the mean (ie, a Bland–Altman plot). From this, the mean difference (delta), which quantifies any bias in the angiography‐derived method, and the 95% limits of agreement, are calculated. The limits of agreement (±1.96 SDs) comprise 95% of observed differences and are akin to the 95% CI of a computed, angiography‐derived FFR result or an error range (Figure). The wider the limits of agreement, the larger the method's error and vice versa. Unlike diagnostic accuracy, the limits of agreement are only a function of how accurate a method is. A recent meta‐analysis of 13 studies of angiography‐derived FFR demonstrated impressive diagnostic accuracy (sensitivity, 89%; specificity, 90%), but more‐sobering agreement, with limits of agreement of FFR ±0.14.^20^ This is remarkably similar to FFR computed from computed tomography in the NXT trial (limits of agreement FFR ±0.15).^21^ FFR computed from computed tomography, however, is a noninvasive screening tool, best used to reduce unnecessary invasive catheterization. Arguably, the accuracy “bar” should be set far higher for a test in the catheter laboratory, where results directly influence decisions regarding proceeding to percutaneous or surgical intervention. Is FFR ±0.14 accurate enough for interventional decision making? It is likely that noninferiority trials will be used to assess these methods. These should avoid the usual pitfalls and be appropriate in terms of power, significance, analysis protocol, sample size, patient population, and prespecified noninferiority margins. Moreover, it remains to be seen how accurate and reproducible these methods are, beyond academic core laboratories, in the hands of those who will be expected to use these tools (ie, the interventional cardiologist operating in the catheter laboratory).

## Conclusions

Angiography‐derived FFR has the potential to change clinical practice for the considerable benefit of patients by providing routine physiological data, together with coronary anatomy, to provide personalized management and improved clinical outcomes. However, deriving physiology from anatomy is challenging and requires assumptions. Model simplification and physiological assumptions, based on extrapolated or averaged data, are likely to work in the majority of patients. However, much of FFR's success lies in its ability to identify those cases where nonstandard microvascular resistance and/or flow result in discordant physiology and anatomy. It is therefore important that models of angiography‐derived FFR retain the same patient‐specific physiology that separates traditional FFR from angiography, or at least that they highlight which cases require more‐reliable assessment. Operators must understand how accuracy and error are defined in all patient groups. Stringent validation is required to prove that models are accurate and physiologically sound, in the hands of those who will be using them. If this can be achieved, clinicians have the potential to achieve what could be a new level of patient‐specific medicine.

## Sources of Funding

Dr Morris is funded by a Wellcome Trust Clinical Research Career Development Fellowship (214567/Z/18/Z).

## Disclosures

Dr Morris has previously received honoraria (speaking fees) from Abbott UK. Professor Curzen has received unrestricted research grants from HeartFlow and Boston Scientific for the FORECAST and RIPCORD2 trials, respectively. The remaining authors have no disclosures to report.
